# Who Feeds the *Trypanosoma cruzi* Vectors? Systematic Review, Geographic Distribution, and Decision Tree of Blood Meal Sources for Brazilian Triatomines

**DOI:** 10.3390/microorganisms14040879

**Published:** 2026-04-14

**Authors:** Maria Clara Silva, Quezia Moura Oliveira, Alena Iñiguez

**Affiliations:** Laboratório de Parasitologia Integrativa e Paleoparasitologia, Instituto Oswaldo Cruz, FIOCRUZ, Av. Brazil 4365, Rio de Janeiro 21040-360, Brazil; mariclara.12silva@gmail.com (M.C.S.); queziamouraeea@gmail.com (Q.M.O.)

**Keywords:** transmission dynamics, ecology, molecular biology

## Abstract

Chagas disease, caused by *Trypanosoma cruzi*, affects 7 million people. Studying the ecology of triatomine vectors through midgut content analysis allows for infection diagnosis and the identification of blood meal sources (BMSs). Current BMS methodologies are limited by the accuracy of genetic data for local fauna, limiting species identification of hosts involved in parasite transmission. Here, we performed a systematic review on BMSs of *T. cruzi* vectors and showed the geographical distribution by *T. cruzi* lineages and vertebrate orders. We propose a decision tree system combining ecological and taxonomic approaches (EcoTaxDT) to discriminate ambiguous BMS results. The EcoTaxDT was validated using published and new BMS results. The review highlights the growing number of BMS studies and the awareness of host species potentially involved in transmission cycles. In Brazilian studies, EcoTaxDT allowed for taxonomic assignments when genetic identity was insufficient or when identified taxa had no geographical occurrence. New BMS results, validated by EcoTaxDT, showed triatomines feeding on *Natalus macrourus*, Echimyidae, Tettigoniidae, and *Tropidurus itambere*. Reliable BMS data and *T. cruzi* diagnosis are crucial for understanding transmission dynamics and human infection risk. EcoTaxDT is functional in correcting inconsistent BMS outputs, ensuring robust and consistent results by integrating genetic, taxonomy, and species geographical distribution.

## 1. Introduction

*Trypanosoma cruzi* Chagas, 1909 (Trypanosomatida, Trypanosomatidae) is a hemoflagellate parasite that causes Chagas disease (CD), a neglected tropical disease, endemic in 21 countries in Latin America [1,2]. Currently, 100 million people are considered at risk of infection, with approximately 7 million individuals infected with the protozoan, more than 10,000 deaths per year, and an estimated annual burden of more than 275,000 disability adjusted life years (DALYs) [2,3]. Despite decades of research, CD continues to pose major medical and social challenges, largely due to limited advances in prevention and treatment [4,5,6,7].

The two most important routes of transmission (vector-borne and vectorial-oral) depend on contact with infected excreta of blood-sucking insects from the Triatominae Jeannel, 1919 (Hemiptera, Reduviidae) subfamily, with intact mucosa or skin lesions, generated after the insect feeding [8,9,10]. Primarily, CD is a zoonosis named American trypanosomiasis with more than 150 mammal species identified as naturally infected with *T. cruzi* in the Americas [11]. In addition, recently, species of reptiles and a bird, taxa historically known as refractories, were found to be *T. cruzi*-infected, raising questions about their role in the dynamics cycle [5,12,13]. Up to this point, 158 species of 19 genera of triatomines are accepted, and all are potentially capable of being infected with *T. cruzi* [14]. The protozoan is genetically classified into seven Discrete Typing Units (DTUs) named TcI-TcVI and TcBat [15,16,17].

The analysis of intestinal content of triatomines can be used for the diagnosis of *T. cruzi* infection and the identification of blood meal sources (BMS). This integrative analysis provides key information for the understanding of ecological relationships in the transmission cycle, detecting host exchange processes, parasite transmission opportunities between local species, and risk of infection to humans [18,19,20]. In addition, BMS studies contribute to characterizing the local fauna by identifying potential hosts and refractory animals that support the vector cycle. BMS research reflects the local specificities on *T. cruzi* network transmission that must be considered for the development and implementation of vector surveillance and control measures [7,20,21].

Studies with populations of *Triatoma brasiliensis* Neiva, 1911 from Rio Grande do Norte state, Northeast Brazil, demonstrated the presence of high rates of *T. cruzi* infection when exhibited as BMS rodents of the Caviidae family, such as *Kerodon rupestris* (Wied-Neuwied, 1820), the Rock Cavy, and *Galea spixii* (Wagler, 1831), the Spix’s Yellow-toothed Cavy [22,23,24,25,26]. These rodents play an important role in maintaining colonies, representing up to 70% of the food sources, and allowing the overlap between wild and peridomestic environments [24].

The identification of BMSs also highlights the presence of domestic animals as important keys for maintaining the infection [20,27,28,29]. Dogs as BMSs may contribute to the persistence of the colonies and infection inside the domicile, being a risk factor for transmission to humans [20,30].

Studies that employ High-Throughput Sequencing (HTS), also known as New Generation Sequencing (NGS), have effectively cleared the challenge of identifying numerous BMS, compared to standard sequencing [31]. For example, Dumontiel and colleagues (2020) were able to demonstrate up to eight different BMSs in one single specimen of *Triatoma sanguisuga* (LeConte, 1856) [18]. Nevertheless, its applicability is limited by the reference sequences available for the specific loci targeted [20,30]. In the absence of genetic deposits in databases, results of unknown BMSs impose significant limitations on the resolution of biodiversity assessments, leading to less specific results [30,32,33,34,35].

This scarcity of genetic data is further evidenced by the limited representation of taxonomic groups of wild vertebrates. In Brazil, gaps in Brazilian fauna species sequences in molecular databases were demonstrated, and associated with biome, traits, diets, taxonomic groups, and threats [36]. This may lead to results with low genetic identity, thereby limiting the specificity of the outcome [37]. Consequently, the occurrence of non-native vertebrate species can be observed in the analysis of BMSs obtained from the intestinal contents of triatomine insects [37,38,39]. For instance, Kieran et al. (2017) reported *Coendou* sp. as one of the two most frequent BMS in the collected *Rhodnius pallescens* Barber, 1932 samples from Panama, in which *Coendou bicolor* (Tschudi, 1844) is the result obtained in GenBank comparison [30]. However, *C. bicolor* is absent in the country, with *Coendou rothschildi* the most likely result of the BMS [30].

In this way, the accuracy of species discrimination of BMS, through molecular biology techniques, relies heavily upon the availability of representative genetic sequences from the local fauna of the study area and the correct taxonomic designation of the genetic deposits in GenBank [40]. As stated by NCBI Taxonomy itself, the platform is not an authoritative source for nomenclature or classification, despite the efforts to make their data more reliable, accessible, and operational [41]. Therefore, taxonomic verification of the results should be conducted by consulting an authoritative taxonomic dataset specifically designed for this purpose, for example, the Integrated Taxonomic Information System (ITIS) (https://www.itis.gov/#), a public database of authoritative taxonomic information on species of the world.

Databases such as the Taxonomic Catalog of the Fauna of Brazil (CTFB = *Catálogo Taxonômico da Fauna do Brasil*) http://fauna.jbrj.gov.br/fauna/listaBrasil/PrincipalUC/PrincipalUC.do?lingua=pt (accessed 19 February 2026) and the Brazilian Biodiversity Information System (SiBBr = *Sistema de Informação sobre a Biodiversidade Brasileira*) https://www.sibbr.gov.br/ (accessed 19 February 2026)are the Brazilian initiatives with this goal, which contribute with important efforts to catalog Brazilian fauna and to identify species occurrence, serving as valuable tools for the curation of geographic distribution data.

The concept of a Decision Tree refers to a structure aimed at minimizing the cost of classifying an object by determining rules [42]. In order to overcome the obstacles observed in species discrimination within studies of insect vector feeding habits, we propose a systematic method for exploring inconsistent and incoherent BMS outcomes with the local fauna of the study region, which we named the Ecological and Taxonomic Decision Tree (EcoTaxDT).

The present study aimed to conduct a systematic review of the literature on triatomine BMSs, as well as an analysis of the geographical distribution of the published findings. In addition, we propose and apply the methodology of EcoTaxDT, demonstrating the validation through BMS results of both already published and new results first published in this present work. We demonstrated the applicability of EcoTaxDT, especially for inconclusive outcomes based on the geographical distribution of GenBank species obtained as initial results.

## 2. Materials and Methods

### 2.1. Systematic Review

The systematic literature review to identify studies that identified BMSs from triatomines through molecular biology methods was performed following the PRISMA 2020 Statement [43]. The PubMed, Scopus, and ScienceDirect databases were searched using Boolean queries with the following keywords (triatomine) AND (“food source” OR “feeding source” OR “blood meal” OR “bloodmeal”) with a time span from 2010 to 2025 (Accessed on 10 November 2025), with reports not in English, reviews and book chapters filtered out. Relevant studies that were not identified through database searches using predefined keywords were manually added.

**Figure 1:**
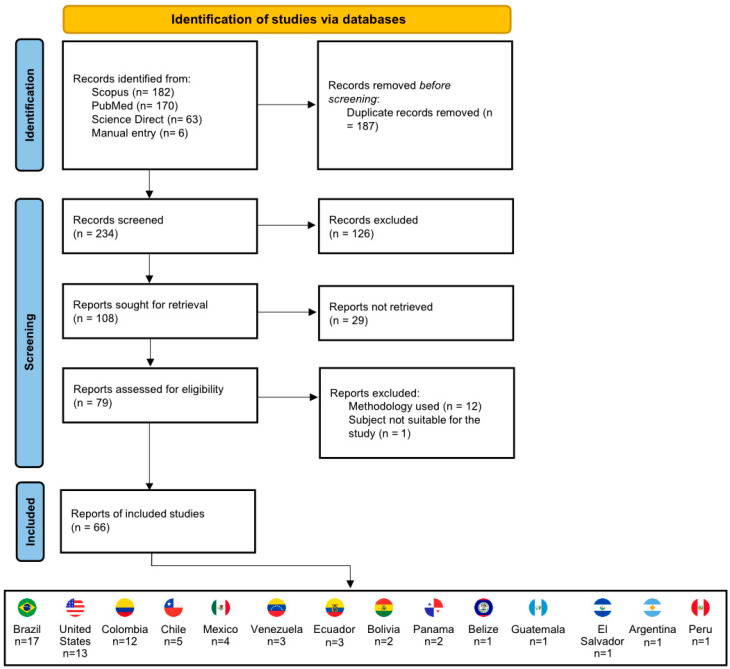
Prisma flow diagram of the systematic literature review of BMS from triatomines through molecular biology methods. The Prisma flow diagram was constructed following Haddaway, N. R., Page, M. J., Pritchard, C. C., & McGuinness, L. A. (2022) [43].

All records were compiled into a Microsoft Excel 2019 spreadsheet (Microsoft Corporation, Redmond, WA, USA), with duplicate entries removed using a software tool and subsequently verified manually. Records were screened by title, and those that did not align with the scope of the review were excluded. Subsequently, the abstracts were analyzed, and the eligible reports were selected for full-text retrieval. For eligibility, only studies that analyzed BMSs through Sanger sequencing or HTS from triatomines collected from the field were accepted (Figure 1 and Table S1).

After the selection of the total included studies, we accessed the country of the study, species of triatomines analyzed, BMS, and *T. cruzi* infection diagnosis (Table S2). Only the studies that were conducted in Brazil were chosen for validation of the EcoTaxDT methodology. This review was not registered in PROSPERO or any other systematic review registry, which should be considered a methodological limitation. A full PRISMA 2020 checklist is available in Figure S1.

### 2.2. Molecular Characterization of Triatomine BMS and T. cruzi Infection

The specimens submitted to molecular identification of the BMS and *T. cruzi* were: *Triatoma brasiliensis* Neiva, 1911 (Hemiptera, Reduviidae) collected in the municipality of Currais Novos, state of Rio Grande do Norte (RN), between the years 2014–2016; *Triatoma pseudomaculata* Correa and Espínola, 1964 (Hemiptera, Reduviidae) collected in the Arcoverde municipality, Pernambuco (PE), in August 2019; and *Triatoma wygodzinskyi* Lent, 1951 from the Vargem Grande do Sul municipality, São Paulo state (SP), collected in June 2023.

The DNA extraction of the intestinal contents occurred after dissection of the triatomines for intestinal content removal using the DNeasy Blood & Tissue Kit (Qiagen, Hilden, Germany) with the following modifications: samples were subjected to digestion with Proteinase K (20 mg/µL) (Invitrogen, Paisley, Scotland), plus 180 µL of Buffer ATL (Tissue Lysis Buffer), and incubated at 56 °C for 12–24 h under stirring at 500 rpm [19]. The DNA concentration was estimated using a Quantus™ Fluorometer (Promega, Madison, WI, USA). The molecular detection of BMSs was based on the DNA Barcoding approach using the genetic markers 12S rDNA (215 bp) [44] and *cyt*b (358 bp) [45]. To detect *T. cruzi* infection and assignment of DTU lineages, the 18S rDNA (350 pb) [46] and *cyt*b (200 pb) [19] molecular targets were applied. The reactions were carried out as described in the manuals of GoTaq^®^ G2 DNA Polymerase (Promega, Madison, WI, USA) or GoTaq^®^ Green Master Mix (Promega, Madison, WI, USA), in the Mastercycler personal Eppendorf ^®^ thermal cycler. PCR products were subjected to 2% agarose gel electrophoresis (Sigma-Aldrich Corporation, St. Louis, MO, USA), stained with GelRed Nucleic Acid Gel Stain (Biotium, Fremont, CA, USA), and visualized under UV light. The amplicons were purified with the ExoSAP-IT™ PCR Product Cleanup Reagent kit (Thermo Fisher Scientific, Carlsbad, CA, USA) and then submitted to DNA direct sequencing using the BigDye Terminator v3.1 Cycle Sequencing Kit (Thermo Fisher Scientific, Carlsbad, CA, USA) and the ABI 3730 sequencer at the RPT01A/FIOCRUZ sequencing facility. The obtained sequences were analyzed and edited by BioEdit v.7.0.9 (Department of Microbiology, North Carolina State University, Raleigh, NC, USA) and Lasergene SeqMan™ v.7.0 (DNASTAR, Madison, WI, USA) programs, where they were then compared by the Basic Local Alignment Search Tool (BLAST) algorithm to the GenBank sequence bank by the National Center for Biotechnology Information platform (NCBI) http://blast.ncbi.nlm.nih.gov/Blast.cgi (accessed 19 February 2026). The cutoff parameters for comparisons using BLAST/NCBI were a genetic identity higher than 95% for species determination and up to 75% for genera and family [22].

### 2.3. EcoTax Decision Tree Construction and Application

The proposed EcoTaxDT is based on 3 main steps: (A) identifying the result obtained from BLAST/NCBI algorithm comparison; (B) accessing taxonomic hierarchy and performing taxonomic validation through ITIS; (C) proceeding with ecological analysis based on the geographical occurrence of the taxon through the CTFB and SiBBr.

**Figure 2:**
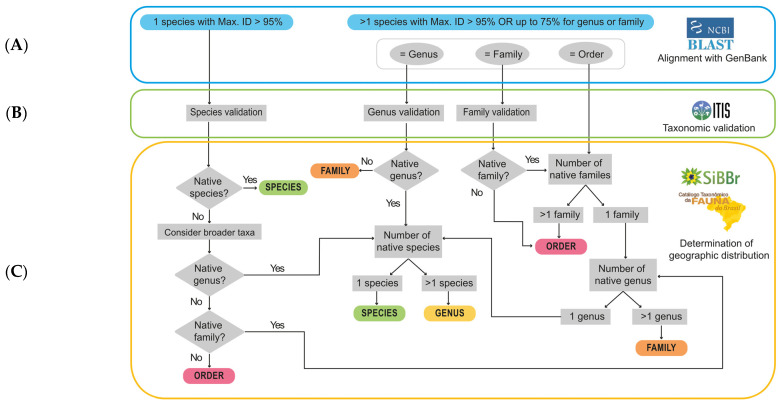
Scheme of the proposed EcoTaxDT. (**A**) Identification of the result obtained from the BLAST/NCBI algorithm comparison. (**B**) Validation of taxonomic status and hierarchy through ITIS (Integrated Taxonomic Information System). (**C**) Ecological analysis based on the geographical occurrence of the taxon through the CTFB (*Catálogo Taxonômico da Fauna do Brasil* = Taxonomic Catalog of the Fauna of Brazil) and SiBBr (*Sistema de Informação sobre a Biodiversidade Brasileira* = Brazilian Biodiversity Information System). The gray diamonds represent decision points, with yes/no questions regarding the occurrence of the taxon in the study location. The gray rectangles indicate verification steps of the number of taxa at a given taxonomic level, with geographic distribution assigned for the study location. The colors at the end of each path represent the taxonomic level of BMS identification: green for species, yellow for genus, orange for family, and red for order. Max. ID: The maximum genetic identity result when compared to GenBank through BLAST/NCBI.

According to the parameters chosen for the study, we defined 2 main scenarios after comparison with the BLAST/NCBI algorithm. In the first case, only 1 species presents the best hits with a genetic identity higher than 95%. In the second case, more than one species presents the best hits, or the value of the maximum genetic identity obtained from alignments is lower than that considered for species-level definition (95%). In both cases, first of all, we validate taxonomic status and hierarchy. In the case of more than 1 species presenting the best hits, it is necessary to identify the lowest taxon in common. Finally, we proceed with the ecological analysis based on the geographical occurrence of the taxon, ensuring that the identified taxa can be present in the study location. When a taxon is not native, we move to broader taxa, for example, from species to genus. The same methodology can be applied to taxa not described in the tree as suborder, infraorder, and subfamilies as additional steps (Figure 2).

## 3. Results

### 3.1. Systematic Review: T. cruzi Diagnosis, Sequencing Methods, and Triatomines Assessment

From an initial total of 421 reports identified, 234 remained for screening after the removal of duplicates. Of these, 79 records were assessed for eligibility, and 66 studies were considered relevant and then included in the review [7,11,18,19,20,22,23,25,26,27,30,31,32,33,34,35,37,38,39,47,48,49,50,51,52,53,54,55,56,57,58,59,60,61,62,63,64,65,66,67,68,69,70,71,72,73,74,75,76,77,78,79,80,81,82,83,84,85,86,87,88,89,90,91,92,93,94]. Among these, 17 studies were conducted with samples collected in Brazil [19,22,23,25,26,27,37,38,39,49,56,59,64,68,69,70,76], and therefore, their results of BMS were analyzed under the EcoTaxDT methodology (Figure 2). Studies that employed Sanger or HTS but utilized restricted primers were not accepted [95,96].

Among the studies included in the review since the first record retrieved in 2012, 40 employed Sanger sequencing for BMS identification, 23 used HTS, with the first publication applying this method in 2017, and 3 applied both methodologies. Since 2020, 51 studies have been published, corresponding to 77.28% of all studies that analyzed triatomine BMS to date.

**Figure 3:**
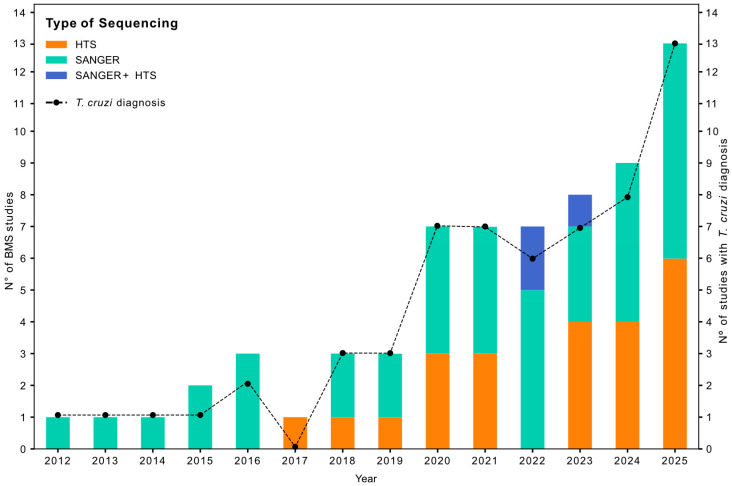
Yearly number of BMS studies by sequencing methods and assessment of *T. cruzi* infection.

**Figure 4:**
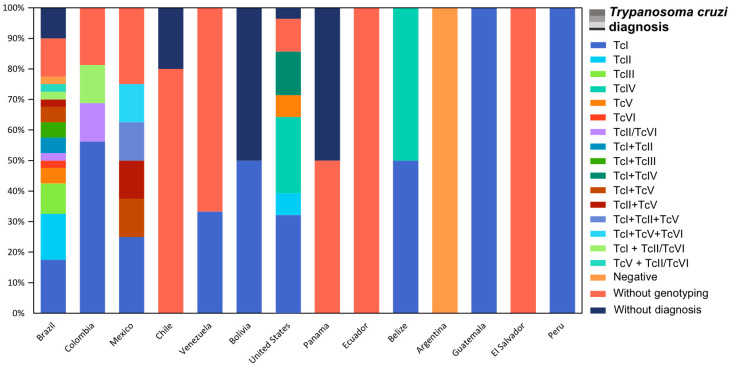
Results of *T. cruzi* diagnosis reported in BMS studies in each country.

Regarding *T. cruzi* infection diagnosis, 58/66 studies also detected *T. cruzi* in naturally collected triatomines, and 34 of these studies additionally performed parasite genotyping (Figure 3). The DTU TcI was the only lineage identified in all studies. Brazil was the country with the most DTUs identified, TcI, TcII, TcIII, TcIV, TcV, and TcVI, more mixed infections, and the only one with the circulation of TcIII. The TcBat lineage was not identified in any of the studies analyzed (Figure 4).

In Brazil, 11 triatomine species had their BMS investigated using sequencing-based methodologies. These species are *Triatoma braziliensis*, *Rhodnius montenegrensis*, *Triatoma arthurneivai*, *Pantrongylus megistus*, *Triatoma pseudomaculata*, *Triatoma petrocchiae*, *Rhodnius nasutus*, *Triatoma sordida*, *Triatoma sherlocki*, *Triatoma melanica,* and *Panstrongylus lutzi*. Among them, *Triatoma brasiliensis* is the species with the greatest number of studies, being included in 9/17 papers in the country (Table S2).

### 3.2. Geographical Distribution of Triatomines BMS

**Figure 5:**
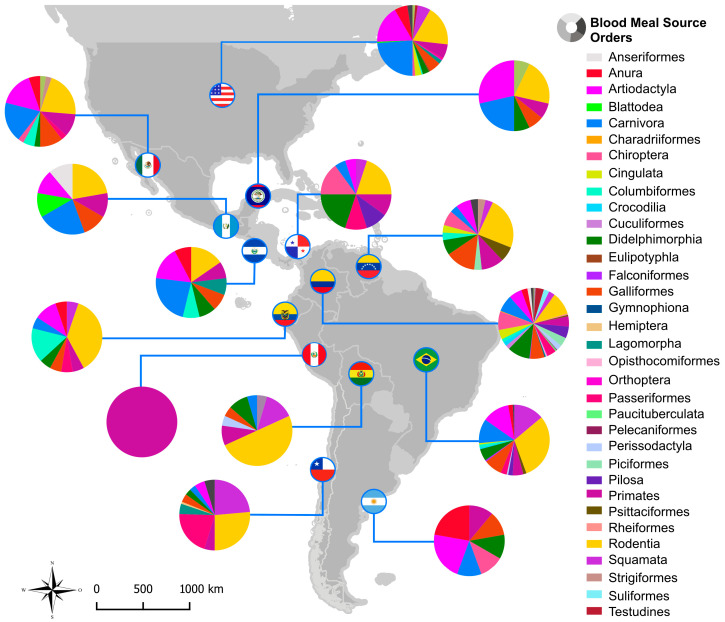
Representative map of taxonomic orders identified as triatomine BMSs in each country. Pie charts illustrate the relative frequency of taxonomic orders identified per country.

The results of the BMS from the systematic review and from the present study showed 34 different orders already identified as BMS in 14 countries (Figure 5). Most frequently identified orders are Rodentia, Carnivora, and Artiodactyla. Brazil, Colombia, and the United States presented the highest diversity of orders already identified, and they are also the countries with the most studies using standard sequencing or HTS to discriminate BMSs.

### 3.3. EcoTaxDT Validation Based on the Systematic Review of the Literature

The systematic review of the molecular definition of triatomine BMSs resulted in 17 articles from Brazil. After analyzing the BMS results, a total of nine results from five articles were selected as examples of the applicability of the EcoTaxDT methodology, based on the value of the genetic identity obtained from GenBank comparison through the BLAST/NCBI algorithm. Detailed analyses are described in Table 1 and Table S3.

The species *Pauxi pauxi* (Linnaeus, 1766) (Cracidae, Galliformes), known as helmeted curassow, was indicated as a BMS for *Rhodnius montenegrensis* in a study conducted in the Amazonas and Rondônia states, North Brazil [39]. This bird is known to have a geographic distribution limited to Venezuela and Colombia [97]. Only the species *Pauxi tomentosa* (Spix, 1808) and *P. tuberosa* (Spix, 1808) have occurrences in the two Brazilian states. In this way, because two species are considered native, the result of BMSs should be discriminated as *Pauxi* genus Temminck, 1813.

In another study, the bird species *Dubusia taeniata* (Boissonneau, 1840) (Thraupidae, Passeriformes), known as the buff-breasted mountain tanager, was referred to as a BMS with a genetic identity of 95% in the RN state [64]. According to SiBBr, members of this genus are not native to the study area. Therefore, following the taxonomic hierarchy, we identified that the family Thraupidae presents 16 native genera for the work area. In this way, according to EcoTaxDT, the result of BMS should be considered as Thraupidae.

*Leopardus geoffroyi* (d’Orbigny and Gervais, 1844) (Felidae, Carnivora), the Geoffroy’s cat, was pointed out as a BMS of *Triatoma sherlocki* collected in Bahia state. However, the feline is not a native species of the study area. According to SiBBr, *Leopardus pardalis* (Linnaeus, 1758) and *L. tigrinus* (Schreber, 1775) have a distribution within the area of the work. Because of that, the result should be defined as genus *Leopardus* Gray, 1842. Another result from the same work identifies, with 93% genetic identity, *Thrichomys inermis*, a species of rodent known as highlands punare, which is native to Bahia state [68]. According to the parameter established for species identification, the result should be consistent and define BMS as genus *Thrichomys* Trouessart, 1880. The same logic should be applied to *Tropidurus hispidus,* which presents a maximum genetic identity of 93%, so the result of BMS could only be defined as a genus or family according to established cut-off agreements. Therefore, solving the BMS result as a member of the native genus *Tropidurus* Wied-Neuwied, 1824.

Valença-Barbosa and coauthors (2022) affirmed that the species *Mustela sibirica* Pallas, 1773 (Mustelidae, Carnivora), the Siberian weasel, is a BMS of *Triatoma melanica* from Minas Gerais state [38]. The species is not native to the study region, and no members of the genus are registered [38]. Therefore, we evaluated the family Mustelidae, which presents, according to CTFB, three native genera, each with only one species in the study area. In this way, the result of BMS should be defined as a member of Mustelidae G. Fischer von Waldheim, 1817.

From another study, we identified three results with genetic identity lower than 95% [22]. *Proceratophrys boiei* (Wied-Neuwied, 1824), the horned frog or leaf frog, is a species of amphibian belonging to the family Odontophrynidae. According to SiBBr, this species is distributed in the South, Southeast, and North regions, but it did not present distribution in the RN state, where the study was conducted. The only species that presents a register of occurrence in the estate is *Proceratophrys cristieps*, but because of the low genetic identity of 83%, the result would be discriminated as genus *Proceratophrys* Miranda-Ribeiro, 1920. The rodent known as Spix’s yellow-toothed cavy, *Galea spixii*, is considered a native species, but because of the low genetic identity of 88%, the best approach is to define the result as a genus *Galea* Meyen, 1832. The result of BMSs on *Mabuya spinalis* was also proposed with a genetic identity of 79%. However, according to the deposit on GenBank (AF280276) that was identified in the study, the species is a synonym of *Chioninia spinalis* (Boulenger, 1906), the current valid species. Thus, according to SiBBr, *C. spinalis* is not a native species, with no records of geographic distribution for the genus in the RN state. Considering that the family Scincidae Oppel, 1811 has a geographic occurrence in the region, the result should be identified as Scincidae Oppel, 1811.

### 3.4. Molecular Characterization of Triatomine BMS, EcoTaxDT Application and T. cruzi Diagnosis

Within the results obtained as BMSs from the triatomines collected, we select four cases (Table 2 and Table S3) to demonstrate and validate the methodology (Figure 2). From an insect collected in the RN state, the bat species *Natalus stramineus* Gray, 1838 (Natalidae, Chiroptera), known as the Brazilian funnel-eared bat, and *Natalus macrourus* (Gervais, 1856), the Brazilian funnel-eared bat, demonstrated a maximum genetic identity of 100.00% (AF345924 and OR879255) [98]. Despite this result, *N. stramineus* is not a species found in the Brazilian Northeast. According to SiBBr and CTFB, the species *N. macrourus* (Gervais, 1856) is the only species of the genus with recorded occurrences in the RN state. For this reason, following what is proposed in the EcoTaxDT, the BMS result was considered as *N. macrourus*.

The second case is from another *T. brasiliensis* specimen from the RN state, with *Clyomys laticeps* (Thomas, 1909) (Echimyidae, Rodentia), the broad-headed spiny rat, as a BMS with a maximum genetic identity of 99.00% (KJ742597). According to the CTFB database, this species is not native to the state, with geographic occurrence in the states of the Midwest and Southeast Brazilian regions, and only in the Bahia state of the Northeast region. Therefore, following the EcoTaxDT, we accessed the members of the Echimydae that present occurrence for the RN state, which returned an uncertain distribution of the family. However, according to SiBBr, there are records of occurrence for two different species of the genus *Thrichomys* Trouessart, 1880, *T. laurentius* Thomas, 1904, and *T. apereoides* (Lund, 1839). Therefore, we concluded that this specimen feeds on a rodent of the Echimydae Gray, 1825.

A single specimen from the PE state presented alignment with the 12S rDNA marker in the BLAST/NCBI with a maximum genetic identity of 95.65% only with a sequence of the species *Cocconotus wheeleri* Hebard, 1927 (Orthoptera, Tettigoniidae) (OR865801). This species is not native to Brazil, presenting reports of occurrence only for Panama [99]. On the SiBR and CTFB databases, there are also no occurrences in Brazil for the *Cocconotus* genus. According to the CTFB, Tettigoniidae Krauss, 1902, known as long-horned grasshoppers, have 11 genera with 12 species considered native to the PE state. Therefore, following the EcoTaxDT, we determined the BMS result as a member of the Tettigoniidae Krauss, 1902.

Finally, the sequences obtained from a specimen of *Triatoma wygodzinskyi* compared to Genbank through the BLAST/NCBI algorithm, showed maximum genetic identity of 95.70% with *T. psammonastes* M. T. Rodrigues, Kashara and Yoneaga-Yassuda, 1988. However, *T. psammonastes* is restricted to a region of Bahia state. Due to the value of the maximum genetic identity being in the cut-off defined for species-level discrimination, we also verified the geographic distribution for other native species of the genus with their genetic sequence deposits available. According to the geographic distribution of this genus, *T. itambere* M. T. Rodrigues, 1987, with 95.62%, exhibits a distribution in the SP state. In addition, *T. torquatus* (Wied-Neuwied, 1820) is also native to the study region and has 89% of genetic identity. Therefore, it cannot be considered as a possible result. Thus, we discriminated the BMS of *T. wygodzinskyi* from the SP state as *T. itambere*.

Molecular diagnosis of *T. cruzi* infection was negative for the specimens of *T. pseudomaculata* and *T. wygodzinskyi* collected in the PE and SP states, respectively. In contrast, both *T. brasiliensis* specimens from the RN state tested positive for *T. cruzi*. Regarding genotyping, DTU TcI was identified in one of the positive samples, whereas the other could not be typed due to a mixed-infection profile observed in the sequencing data.

## 4. Discussion

Reliable *T. cruzi* diagnosis and BMS data are crucial for understanding transmission dynamics and assessing human infection risk. The EcoTaxDT methodology proposed here serves as a validation step in BMS studies, when the results show a low genetic identity or when the identified species has no geographical occurrence, ensuring reproducible results by integrating genetic and taxonomy with species distribution and correcting inconsistent outputs. This accurate BMS definition, which is informative of the potential *T. cruzi* hosts and fauna involved in the vector cycle, contributed to a better understanding of parasite transmission networks at the local level.

The systematic review of the literature highlights the increase in publications from 2020 to date employing direct sequencing and HTS. This trend does not indicate a reduction in studies using direct sequencing, but rather a pronounced increase in those applying HTS methodologies. These approaches are increasingly becoming more affordable, cost-effective, and the produced data is easier to analyze due to the development of powerful bioinformatic tools that simplify the processing [100]. These could explain the increase in the studies published with this methodology in recent years. As already suggested, the advent of HTS technologies has enormous potential for the simultaneous understanding of the components involved in the transmission cycles of vector-borne pathogens (e.g., vertebrate blood-meal sources, midgut microbiome composition, pathogen diversity, and vector diversity) [100]. Presenting advantages for identifying mixed BMS, these methods not only reveal a vast diversity of hosts but also increase the number of taxa detected per individual insect [47].

In our search, studies that utilized other methodologies for BMS identification, such as Enzyme-Linked Immunosorbent Assay (ELISA) and High-Resolution Melting (HRM), were also identified. Methodologies based on antigen–antibody reaction were among the first approaches used for BMS identification; however, they exhibited low sensitivity and specificity, often failing to accurately reflect the true diversity of BMS [19]. In contrast, the HRM technique offers certain advantages, such as higher sensitivity and faster processing time. Conversely, it also has restrictions, including limited taxonomic resolution and dependence on prior knowledge of the target [101]. This methodology was applied individually to identify BMS [102,103] and, in some studies, combined with sequencing-based approaches to resolve inconclusive melting curve results, enabling the identification of non-preselected fauna [11,94]. Brazil is the country with the highest number of BMS studies and the second highest in BMS diversity, with Rodentia, Squamata, and Artiodactyla as the main orders identified. Of the 64 triatomine species recorded in the country [14], only 11 had their BMS analyzed using sequencing-based methodologies. Among the 17 studies identified, 9 were conducted with *T. brasiliensis,* and 11 were carried out in the Northeast region of Brazil. The semi-arid region of Brazil remains very important for CD, especially due to the presence of *T. brasiliensis*, considered the most epidemiologically important vector due to adaptation to human-modified environments and elevated rates of *T. cruzi* infection [23,25,26]. The significance of this vector is highlighted by the number of studies, but secondary vectors are also relevant to better understand the cycles in the wild.

Despite the low number of reported CD cases in the USA, most of which are non-native or congenital, a great number of studies have highlighted the potential risk of local transmission and the possibility of infection [48,61]. In our systematic review, the USA presented the second highest number of BMS studies, equal to Colombia, with mammals of Carnivora, Rodentia, and Artiodactyla orders, with the highest frequency and all positive to *T. cruzi* infection. The identification of the risk factor components for parasite transmission in eco-epidemiological studies has emphasized the risk of human transmission, making CD now recognized as an emerging disease in the United States [104].

It is important to underline that the high diversity observed in Brazil, Colombia, and the United States is influenced by both the number of studies conducted and the methodologies applied. In Colombia, for example, the country with the greatest reported diversity of BMS orders, a single study using HTS was able to identify 79 taxa as BMSs of *Rhodnius prolixus* [74]. By analyzing the already published results of triatomine BMS molecular identification, we reinforce the necessity of using the taxonomic hierarchy and the occurrence of the taxa as guides for a conservative, but specific and robust discrimination of species enrolled in the *T. cruzi* transmission net. We recognize that some studies already try to be conservative when sequences present low genetic identity, defining BMS results as genus level [22], and also realize the necessity of assessing the geographic distribution of species [37,38]. However, others do not consider the geographic occurrence of species, as the example mentioned above of the Geoffroy’s cat, *L. geoffroyi*, a species that does not occur in the location of the study where it was identified as a BMS of *T. sherlocki* [68].

We also emphasize the importance of taxon validation by means of reliable sources of information, such as the ITIS database utilized in the present paper. Pires-Silva and coauthors (2025) defined *Dasypus yepesi* and *Phyllomedusa aff. hypochondriali* as BMSs of *Triatoma braziliensis* [26], and according to ITIS, the two taxa are considered invalid, being synonymous with *Dasypus mazzai* and *Pithecopus hypochondrialis*, respectively. It is also important to note that there is no evidence of geographic distribution of *D. mazzai* in Brazil.

Oliveira and coauthors (2025) [40] emphasize that the lack of genetic reference sequences of Brazilian mammals, especially wild *T. cruzi* hosts, for commonly used markers in BMS identification directly compromises the accuracy of species definition, thereby impacting the understanding of parasite transmission dynamics.

It is also imperative that the quality and taxonomic veracity of the already published genetic information be maintained. In the analysis conducted in the present work, we observed that the sequence OP699684, which was deposited as *Triatoma melanica*, in fact presents high identity with a carnivore from the Mustelidae family (MN206976), corresponding to *Galictis cuja* (Lesser Grison) according to the authors [28]. We highlight that these deposits may affect current research if a refined analysis is not performed. We underline the relevance of showing the results of the parameters obtained on BLAST/NCBI or delimited cutoffs for transparency of the work. The cutoff for taxon genetic identification should be defined with caution, depending on the genetic marker utilized and the taxonomic group analyzed. Based on the review conducted here, 37.88% of BMS studies (41/66) do not establish a genetic identification cutoff for species delimitation.

Regarding the limitations of the EcoTaxDT methodology, we highlight that the chosen genetic cutoff for genetic identification was based on the literature of the barcoding DNA approach for BMS identification. However, we acknowledge that for some taxonomic groups, this cutoff may be overly restrictive or inclusive. One more limitation is related to its applicability only to studies that use nucleotide sequencing-based approaches for BMS assessment.

In addition, the verification steps of the EcoTaxDT depend on the curated data deposited in online databases used for taxonomic validation, genetic identification, and geographic verification of occurrence. Geographic occurrence data can be particularly challenging due to the difficulty of sampling many vertebrate taxa in the Neotropics. We acknowledge the efforts of Brazilian databases in updating and curating information. However, we are aware that some taxa are still not fully cataloged. We also recognize the difficulty in accessing reliable occurrence data for many species in other countries.

Insects of Tettigoniidae were described as BMSs of triatomines for the first time in the present study. Other orthopters have already been identified as BMSs [54,63]. The feeding of *T. pseudomaculata* on insects was mentioned using the precipitin assay [105]. This result revealed the ability to diversify their diet according to environmental availability, showing how opportunistic these vectors are and how important these animals can be for maintaining infestations. Lizards of the *Tropidurus* genus were here reported as BMSs of *T. wygodzinsky. Tropidurus* species have already been identified as BMSs for *T. brasiliensis*, *T. petrocchiae*, and *T. sherlocki* [23,25,64,68]. Additionally, *T. itambere* has been reported as PCR-positive in *Trypanosoma* sp. infection diagnostics [106]. Lizards have been proposed as silent hosts of *T. cruzi* in studies conducted in Chile [11,13].

The identification of bats and rodents, known as classic hosts of *T. cruzi*, as BMSs of *T. brasiliensis* in the present study, corroborates knowledge about this species of vector and its importance for transmission cycles regarding possible exposure to infection.

The methodology proposed should be utilized as a final step in the study of vector BMSs, as a way of ensuring the most accurate result supported by the genetic identification and geographical distribution of species in a systematic approach, guaranteeing the reproducibility of the analysis. Furthermore, it allows the exploration of incoherent genetic results with a more consistent taxonomic assignment.

Our results highlight the necessity of applying a systematic approach to support the identification of faunal species in the absence of genetic deposits for Brazilian local fauna in GenBank, particularly in BMS studies of Chagas disease vectors. Finally, the EcoTaxDT methodology could be applied to refined results of BMS species from other countries endemic to Chagas disease using the corresponding fauna database of authoritative taxonomic information on local species.

## Figures and Tables

**Table 1:** Results of the BMS definition before and after the application of the EcoTaxDT system in triatomine samples from the literature.

Literature BLAST/NCBI Result	Max. ID	GenBank Accession Number	BMS Taxonomic Identification After EcoTaxDT
*Dubusia taeniata* [23]	90.00%	AY383098	Thraupidae
*Galea spixii* [22]	88.00%	GU067492	*Galea* sp.
*Leopardus geoffroyi* [68]	95.49%	KP202292	*Leopardus* sp.
*Mabuya spinalis* * [22]	79.00%	AF280276	Scincidae
*Mustela sibirica* [38]	97.26%	AP017396	Mustelidae
*Pauxi pauxi* [39]	92.28%	AF068190	*Pauxi* sp.
*Proceratophrys boiei* [22]	83.00%	KF214166	*Proceratophrys* sp.
*Thrichomys inermis* [68]	93.00%	JX459887	*Thrichomys* sp.
*Tropidurus hispidus* [68]	93.71%	EF616030	*Tropidurus* sp.

Max. ID: The maximum genetic identity result when compared to GenBank through BLAST/NCBI. BMS: Blood meal source. EcoTaxDT: Ecological and Taxonomic Decision Tree. * Invalid taxonomic name considered synonyms with *Chioninia spinalis*.

**Table 2:** Results of the BMS definition before and after the application of the EcoTaxDT system in triatomine samples analyzed in the present study.

Specimen ID	Locality	BLAST/NCBI Result	Max. ID	GenBank Accession Number	BMS Taxonomic Identification After EcoTaxDT
PE05	PE	*Cocconotus wheeleri*	95.65%	OR865801	Tettigoniidae
CN6318	RN	*Clyomys laticeps*	99.00%	KU892753	Echimyidae
CN6260	RN	*Natalus stramineus* *Natalus macrourus*	100.00%	AF345924 OR879255	*Natalus macrourus*
SP06	SP	*Tropidurus psammonastes*	95.70%	KU245079	*Tropidurus itambere*

Specimen ID: Laboratory specimen identification. Locality: Brazilian states. RN: Rio Grande do Norte. PE: Pernambuco. SP: São Paulo. Max.ID: The maximum genetic identity result when compared to GenBank through BLAST/NCBI. BMS: Blood meal source. EcoTaxDT: Ecological and Taxonomic Decision Tree.

## Data Availability

All sequences generated in this study have been submitted to the GenBank database under the accession numbers: PX691516 and PX695482-84. Data will be made available on request.

## Supplementary Materials

The following supporting information can be downloaded at: https://www.mdpi.com/article/10.3390/microorganisms14040879/s1, Figure S1: PRISMA 2020 checklist; Table S1: PRISMA screening, eligibility assessment, and inclusion of studies in the systematic review; Table S2: Data extracted from the literature review; Table S3: Detailed EcoTaxDT application. Reference [107] is cited in the supplementary materials.

## Abbreviations

The following abbreviations are used in this manuscript:

BMS	Blood Meal Source
CD	Chagas Disease
EcoTaxDT	Ecological and Taxonomic Decision Tree
HTS	High Throughput Sequencing
DTU	Discrete Typing Unit
ITIS	Integrated Taxonomic Information System
CTFB	Taxonomic Catalog of the Fauna of Brazil
BLAST	Basic Local Alignment Search Tool
SiBBr	Brazilian Biodiversity Information System
NCBI	National Center for Biotechnology Information
PE	Pernambuco
RN	Rio Grande do Norte
SP	São Paulo
ELISA	Enzyme-Linked Immunosorbent Assay
HRM	High-Resolution Melting
